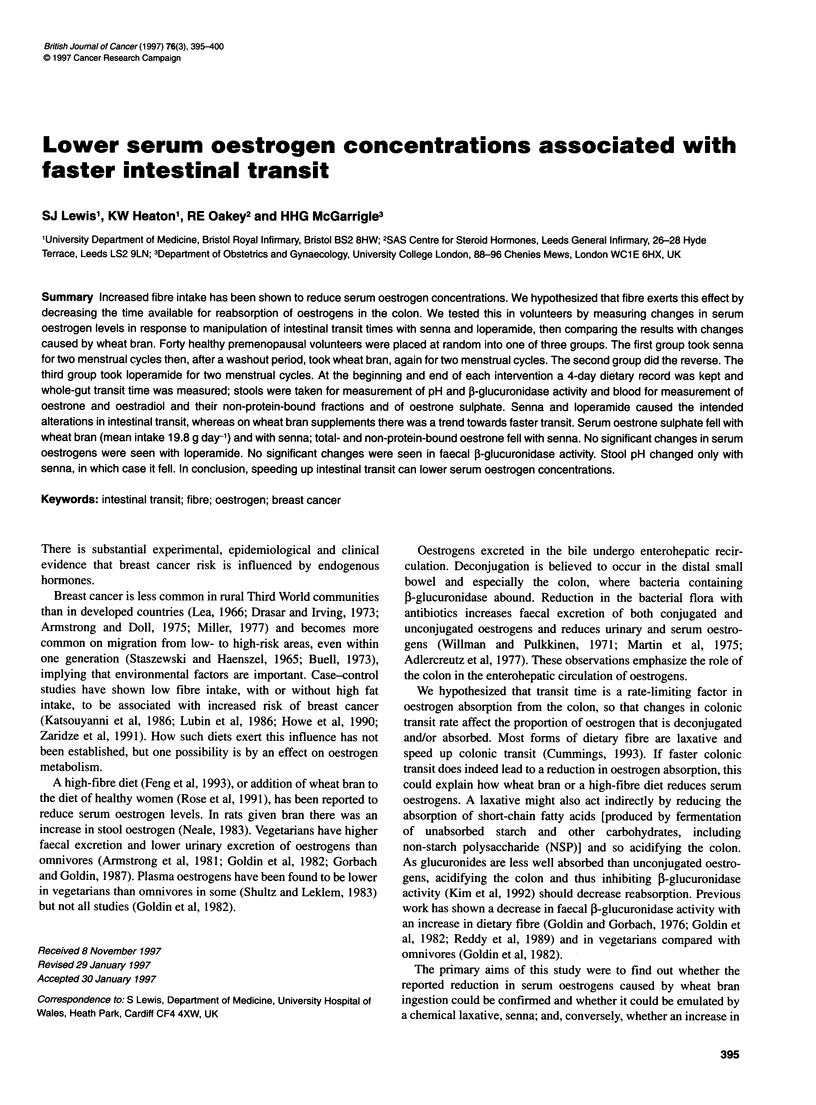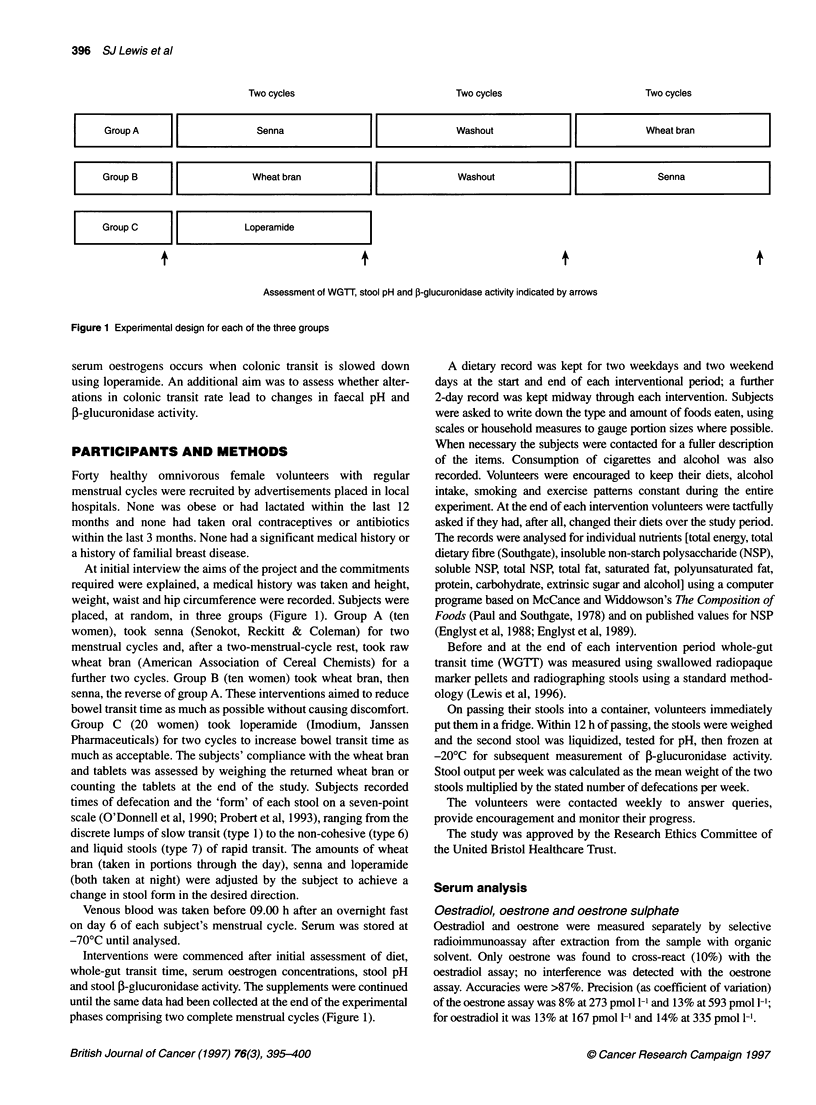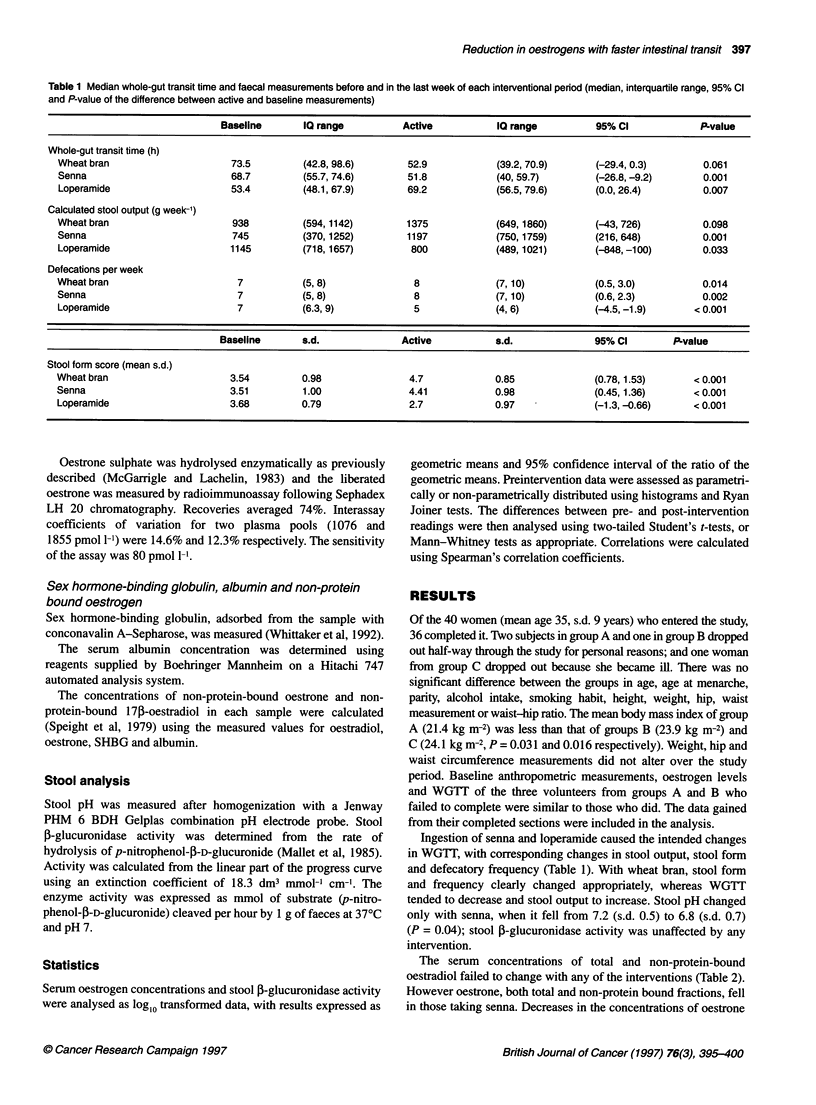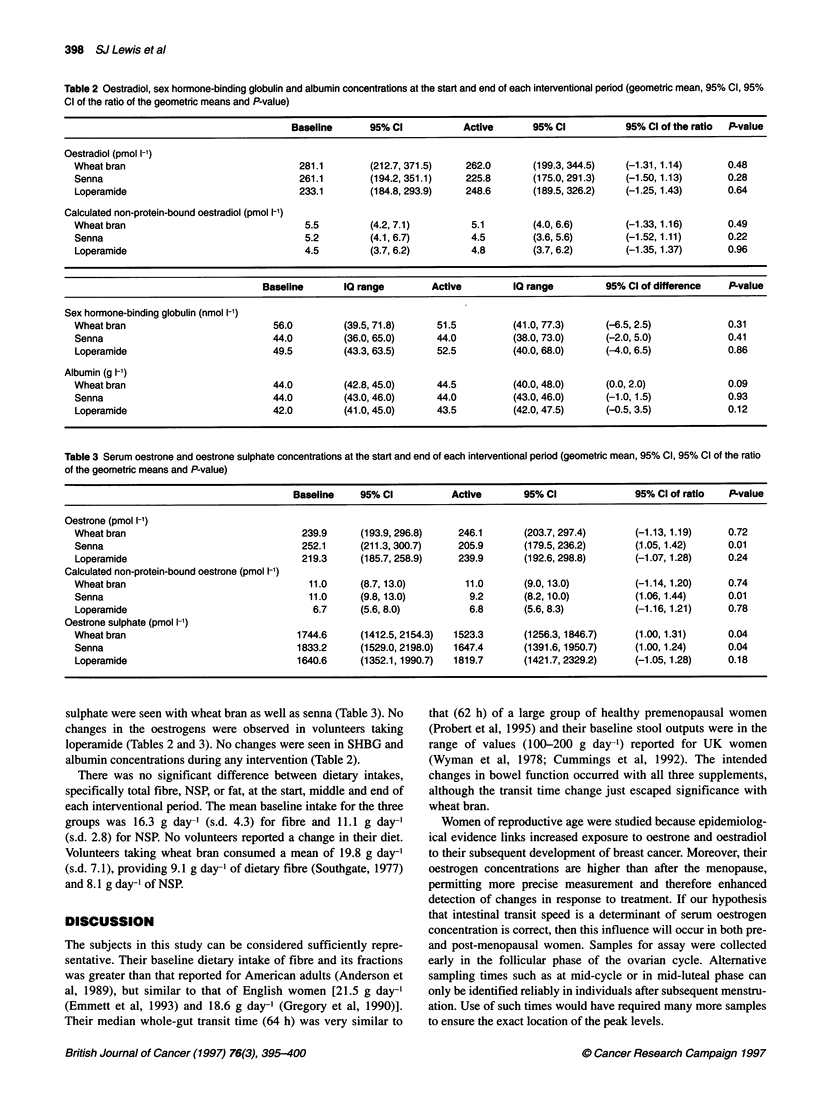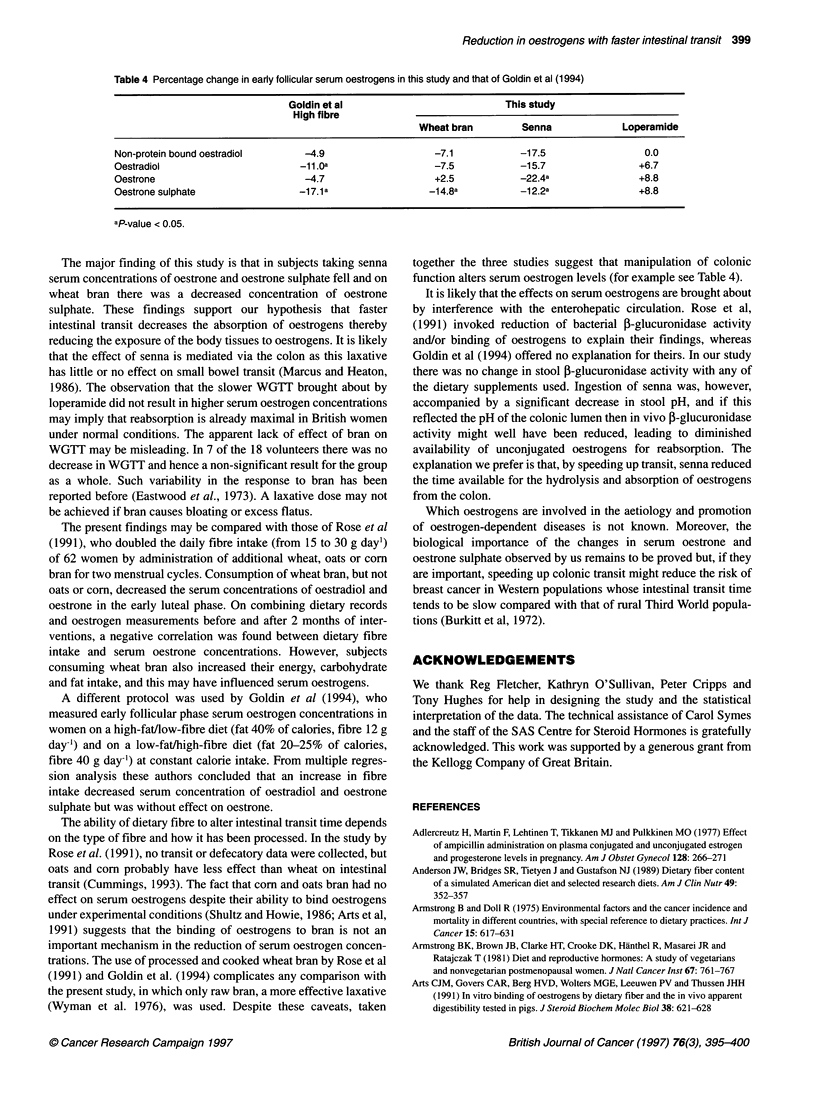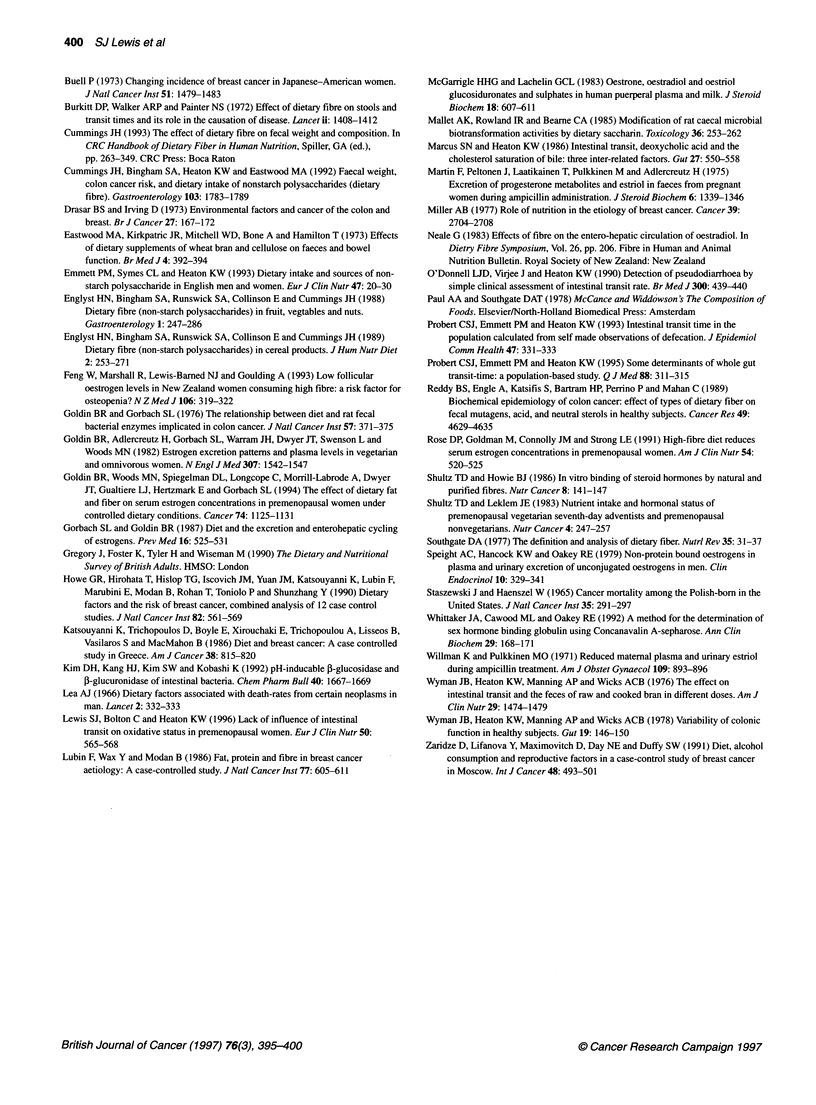# Lower serum oestrogen concentrations associated with faster intestinal transit.

**DOI:** 10.1038/bjc.1997.397

**Published:** 1997

**Authors:** S. J. Lewis, K. W. Heaton, R. E. Oakey, H. H. McGarrigle

**Affiliations:** University Department of Medicine, Bristol Royal Infirmary, UK.

## Abstract

Increased fibre intake has been shown to reduce serum oestrogen concentrations. We hypothesized that fibre exerts this effect by decreasing the time available for reabsorption of oestrogens in the colon. We tested this in volunteers by measuring changes in serum oestrogen levels in response to manipulation of intestinal transit times with senna and loperamide, then comparing the results with changes caused by wheat bran. Forty healthy premenopausal volunteers were placed at random into one of three groups. The first group took senna for two menstrual cycles then, after a washout period, took wheat bran, again for two menstrual cycles. The second group did the reverse. The third group took loperamide for two menstrual cycles. At the beginning and end of each intervention a 4-day dietary record was kept and whole-gut transit time was measured; stools were taken for measurement of pH and beta-glucuronidase activity and blood for measurement of oestrone and oestradiol and their non-protein-bound fractions and of oestrone sulphate. Senna and loperamide caused the intended alterations in intestinal transit, whereas on wheat bran supplements there was a trend towards faster transit. Serum oestrone sulphate fell with wheat bran (mean intake 19.8 g day(-1)) and with senna; total- and non-protein-bound oestrone fell with senna. No significant changes in serum oestrogens were seen with loperamide. No significant changes were seen in faecal beta-glucuronidase activity. Stool pH changed only with senna, in which case it fell. In conclusion, speeding up intestinal transit can lower serum oestrogen concentrations.


British Journal of Cancer (1997) 76(3), 395–400
© 1997 Cancer Research Campaign

### Lower serum oestrogen concentrations associated with faster intestinal transit

**SJ Lewis[1], KW Heaton[1], RE Oakey[2] and HHG McGarrigle[3]**

[1]University Department of Medicine, Bristol Royal Infirmary, Bristol BS2 8HW; [2]SAS Centre for Steroid Hormones, Leeds General Infirmary, 26–28 Hyde Terrace, Leeds LS2 9LN; [3]Department of Obstetrics and Gynaecology, University College London, 88–96 Chenies Mews, London WC1E 6HX, UK

**Summary** Increased fibre intake has been shown to reduce serum oestrogen concentrations. We hypothesized that fibre exerts this effect by decreasing the time available for reabsorption of oestrogens in the colon. We tested this in volunteers by measuring changes in serum oestrogen levels in response to manipulation of intestinal transit times with senna and loperamide, then comparing the results with changes caused by wheat bran. Forty healthy premenopausal volunteers were placed at random into one of three groups. The first group took senna for two menstrual cycles then, after a washout period, took wheat bran, again for two menstrual cycles. The second group did the reverse. The third group took loperamide for two menstrual cycles. At the beginning and end of each intervention a 4-day dietary record was kept and whole-gut transit time was measured; stools were taken for measurement of pH and β-glucuronidase activity and blood for measurement of oestrone and oestradiol and their non-protein-bound fractions and of oestrone sulphate. Senna and loperamide caused the intended alterations in intestinal transit, whereas on wheat bran supplements there was a trend towards faster transit. Serum oestrone sulphate fell with wheat bran (mean intake 19.8 g day$^{-1}$) and with senna; total- and non-protein-bound oestrone fell with senna. No significant changes in serum oestrogens were seen with loperamide. No significant changes were seen in faecal β-glucuronidase activity. Stool pH changed only with senna, in which case it fell. In conclusion, speeding up intestinal transit can lower serum oestrogen concentrations.

**Keywords:** intestinal transit; fibre; oestrogen; breast cancer

There is substantial experimental, epidemiological and clinical evidence that breast cancer risk is influenced by endogenous hormones.

Breast cancer is less common in rural Third World communities than in developed countries (Lea, 1966; Drasar and Irving, 1973; Armstrong and Doll, 1975; Miller, 1977) and becomes more common on migration from low- to high-risk areas, even within one generation (Staszewski and Haenszel, 1965; Buell, 1973), implying that environmental factors are important. Case–control studies have shown low fibre intake, with or without high fat intake, to be associated with increased risk of breast cancer (Katsouyanni et al, 1986; Lubin et al, 1986; Howe et al, 1990; Zaridze et al, 1991). How such diets exert this influence has not been established, but one possibility is by an effect on oestrogen metabolism.

A high-fibre diet (Feng et al, 1993), or addition of wheat bran to the diet of healthy women (Rose et al, 1991), has been reported to reduce serum oestrogen levels. In rats given bran there was an increase in stool oestrogen (Neale, 1983). Vegetarians have higher faecal excretion and lower urinary excretion of oestrogens than omnivores (Armstrong et al, 1981; Goldin et al, 1982; Gorbach and Goldin, 1987). Plasma oestrogens have been found to be lower in vegetarians than omnivores in some (Shultz and Leklem, 1983) but not all studies (Goldin et al, 1982).

*Received 8 November 1997*
*Revised 29 January 1997*
*Accepted 30 January 1997*

*Correspondence to:* S Lewis, Department of Medicine, University Hospital of Wales, Heath Park, Cardiff CF4 4XW, UK

Oestrogens excreted in the bile undergo enterohepatic recirculation. Deconjugation is believed to occur in the distal small bowel and especially the colon, where bacteria containing β-glucuronidase abound. Reduction in the bacterial flora with antibiotics increases faecal excretion of both conjugated and unconjugated oestrogens and reduces urinary and serum oestrogens (Willman and Pulkkinen, 1971; Martin et al, 1975; Adlercreutz et al, 1977). These observations emphasize the role of the colon in the enterohepatic circulation of oestrogens.

We hypothesized that transit time is a rate-limiting factor in oestrogen absorption from the colon, so that changes in colonic transit rate affect the proportion of oestrogen that is deconjugated and/or absorbed. Most forms of dietary fibre are laxative and speed up colonic transit (Cummings, 1993). If faster colonic transit does indeed lead to a reduction in oestrogen absorption, this could explain how wheat bran or a high-fibre diet reduces serum oestrogens. A laxative might also act indirectly by reducing the absorption of short-chain fatty acids [produced by fermentation of unabsorbed starch and other carbohydrates, including non-starch polysaccharide (NSP)] and so acidifying the colon. As glucuronides are less well absorbed than unconjugated oestrogens, acidifying the colon and thus inhibiting β-glucuronidase activity (Kim et al, 1992) should decrease reabsorption. Previous work has shown a decrease in faecal β-glucuronidase activity with an increase in dietary fibre (Goldin and Gorbach, 1976; Goldin et al, 1982; Reddy et al, 1989) and in vegetarians compared with omnivores (Goldin et al, 1982).

The primary aims of this study were to find out whether the reported reduction in serum oestrogens caused by wheat bran ingestion could be confirmed and whether it could be emulated by a chemical laxative, senna; and, conversely, whether an increase in

**Figure 1** Experimental design for each of the three groups serum oestrogens occurs when colonic transit is slowed down using loperamide. An additional aim was to assess whether alterations in colonic transit rate lead to changes in faecal pH and β-glucuronidase activity.

### PARTICIPANTS AND METHODS

Forty healthy omnivorous female volunteers with regular menstrual cycles were recruited by advertisements placed in local hospitals. None was obese or had lactated within the last 12 months and none had taken oral contraceptives or antibiotics within the last 3 months. None had a significant medical history or a history of familial breast disease.

At initial interview the aims of the project and the commitments required were explained, a medical history was taken and height, weight, waist and hip circumference were recorded. Subjects were placed, at random, in three groups (Figure 1). Group A (ten women), took senna (Senokot, Reckitt & Coleman) for two menstrual cycles and, after a two-menstrual-cycle rest, took raw wheat bran (American Association of Cereal Chemists) for a further two cycles. Group B (ten women) took wheat bran, then senna, the reverse of group A. These interventions aimed to reduce bowel transit time as much as possible without causing discomfort. Group C (20 women) took loperamide (Imodium, Janssen Pharmaceuticals) for two cycles to increase bowel transit time as much as acceptable. The subjects' compliance with the wheat bran and tablets was assessed by weighing the returned wheat bran or counting the tablets at the end of the study. Subjects recorded times of defecation and the 'form' of each stool on a seven-point scale (O'Donnell et al, 1990; Probert et al, 1993), ranging from the discrete lumps of slow transit (type 1) to the non-cohesive (type 6) and liquid stools (type 7) of rapid transit. The amounts of wheat bran (taken in portions through the day), senna and loperamide (both taken at night) were adjusted by the subject to achieve a change in stool form in the desired direction.

Venous blood was taken before 09.00 h after an overnight fast on day 6 of each subject's menstrual cycle. Serum was stored at –70°C until analysed.

Interventions were commenced after initial assessment of diet, whole-gut transit time, serum oestrogen concentrations, stool pH and stool β-glucuronidase activity. The supplements were continued until the same data had been collected at the end of the experimental phases comprising two complete menstrual cycles (Figure 1).

A dietary record was kept for two weekdays and two weekend days at the start and end of each interventional period; a further 2-day record was kept midway through each intervention. Subjects were asked to write down the type and amount of foods eaten, using scales or household measures to gauge portion sizes where possible. When necessary the subjects were contacted for a fuller description of the items. Consumption of cigarettes and alcohol was also recorded. Volunteers were encouraged to keep their diets, alcohol intake, smoking and exercise patterns constant during the entire experiment. At the end of each intervention volunteers were tactfully asked if they had, after all, changed their diets over the study period. The records were analysed for individual nutrients [total energy, total dietary fibre (Southgate), insoluble non-starch polysaccharide (NSP), soluble NSP, total NSP, total fat, saturated fat, polyunsaturated fat, protein, carbohydrate, extrinsic sugar and alcohol] using a computer programe based on McCance and Widdowson's *The Composition of Foods* (Paul and Southgate, 1978) and on published values for NSP (Englyst et al, 1988; Englyst et al, 1989).

Before and at the end of each intervention period whole-gut transit time (WGTT) was measured using swallowed radiopaque marker pellets and radiographing stools using a standard methodology (Lewis et al, 1996).

On passing their stools into a container, volunteers immediately put them in a fridge. Within 12 h of passing, the stools were weighed and the second stool was liquidized, tested for pH, then frozen at –20°C for subsequent measurement of β-glucuronidase activity. Stool output per week was calculated as the mean weight of the two stools multiplied by the stated number of defecations per week.

The volunteers were contacted weekly to answer queries, provide encouragement and monitor their progress.

The study was approved by the Research Ethics Committee of the United Bristol Healthcare Trust.

### Serum analysis

#### *Oestradiol, oestrone and oestrone sulphate*

Oestradiol and oestrone were measured separately by selective radioimmunoassay after extraction from the sample with organic solvent. Only oestrone was found to cross-react (10%) with the oestradiol assay; no interference was detected with the oestrone assay. Accuracies were >87%. Precision (as coefficient of variation) of the oestrone assay was 8% at 273 pmol $l^{-1}$ and 13% at 593 pmol $l^{-1}$; for oestradiol it was 13% at 167 pmol $l^{-1}$ and 14% at 335 pmol $l^{-1}$.

© Cancer Research Campaign 1997

**Table 1** Median whole-gut transit time and faecal measurements before and in the last week of each interventional period (median, interquartile range, 95% CI and *P*-value of the difference between active and baseline measurements)

| | Baseline | IQ range | Active | IQ range | 95% CI | *P*-value |
|---|---|---|---|---|---|---|
| Whole-gut transit time (h) | | | | | | |
| Wheat bran | 73.5 | (42.8, 98.6) | 52.9 | (39.2, 70.9) | (–29.4, 0.3) | 0.061 |
| Senna | 68.7 | (55.7, 74.6) | 51.8 | (40, 59.7) | (–26.8, –9.2) | 0.001 |
| Loperamide | 53.4 | (48.1, 67.9) | 69.2 | (56.5, 79.6) | (0.0, 26.4) | 0.007 |
| Calculated stool output (g week$^{-1}$) | | | | | | |
| Wheat bran | 938 | (594, 1142) | 1375 | (649, 1860) | (–43, 726) | 0.098 |
| Senna | 745 | (370, 1252) | 1197 | (750, 1759) | (216, 648) | 0.001 |
| Loperamide | 1145 | (718, 1657) | 800 | (489, 1021) | (–848, –100) | 0.033 |
| Defecations per week | | | | | | |
| Wheat bran | 7 | (5, 8) | 8 | (7, 10) | (0.5, 3.0) | 0.014 |
| Senna | 7 | (5, 8) | 8 | (7, 10) | (0.6, 2.3) | 0.002 |
| Loperamide | 7 | (6.3, 9) | 5 | (4, 6) | (–4.5, –1.9) | < 0.001 |

| | Baseline | s.d. | Active | s.d. | 95% CI | *P*-value |
|---|---|---|---|---|---|---|
| Stool form score (mean s.d.) | | | | | | |
| Wheat bran | 3.54 | 0.98 | 4.7 | 0.85 | (0.78, 1.53) | < 0.001 |
| Senna | 3.51 | 1.00 | 4.41 | 0.98 | (0.45, 1.36) | < 0.001 |
| Loperamide | 3.68 | 0.79 | 2.7 | 0.97 | (–1.3, –0.66) | < 0.001 |

Oestrone sulphate was hydrolysed enzymatically as previously described (McGarrigle and Lachelin, 1983) and the liberated oestrone was measured by radioimmunoassay following Sephadex LH 20 chromatography. Recoveries averaged 74%. Interassay coefficients of variation for two plasma pools (1076 and 1855 pmol $l^{-1}$) were 14.6% and 12.3% respectively. The sensitivity of the assay was 80 pmol $l^{-1}$.

### *Sex hormone-binding globulin, albumin and non-protein bound oestrogen*

Sex hormone-binding globulin, adsorbed from the sample with conconavalin A–Sepharose, was measured (Whittaker et al, 1992).

The serum albumin concentration was determined using reagents supplied by Boehringer Mannheim on a Hitachi 747 automated analysis system.

The concentrations of non-protein-bound oestrone and non-protein-bound 17β-oestradiol in each sample were calculated (Speight et al, 1979) using the measured values for oestradiol, oestrone, SHBG and albumin.

### Stool analysis

Stool pH was measured after homogenization with a Jenway PHM 6 BDH Gelplas combination pH electrode probe. Stool β-glucuronidase activity was determined from the rate of hydrolysis of *p*-nitrophenol-β-D-glucuronide (Mallet et al, 1985). Activity was calculated from the linear part of the progress curve using an extinction coefficient of 18.3 $dm^3$ $mmol^{-1}$ $cm^{-1}$. The enzyme activity was expressed as mmol of substrate (*p*-nitrophenol-β-D-glucuronide) cleaved per hour by 1 g of faeces at 37°C and pH 7.

### Statistics

Serum oestrogen concentrations and stool β-glucuronidase activity were analysed as $\log_{10}$ transformed data, with results expressed as geometric means and 95% confidence interval of the ratio of the geometric means. Preintervention data were assessed as parametrically or non-parametrically distributed using histograms and Ryan Joiner tests. The differences between pre- and post-intervention readings were then analysed using two-tailed Student's *t*-tests, or Mann–Whitney tests as appropriate. Correlations were calculated using Spearman's correlation coefficients.

### RESULTS

Of the 40 women (mean age 35, s.d. 9 years) who entered the study, 36 completed it. Two subjects in group A and one in group B dropped out half-way through the study for personal reasons; and one woman from group C dropped out because she became ill. There was no significant difference between the groups in age, age at menarche, parity, alcohol intake, smoking habit, height, weight, hip, waist measurement or waist–hip ratio. The mean body mass index of group A (21.4 kg $m^{-2}$) was less than that of groups B (23.9 kg $m^{-2}$) and C (24.1 kg $m^{-2}$, $P = 0.031$ and 0.016 respectively). Weight, hip and waist circumference measurements did not alter over the study period. Baseline anthropometric measurements, oestrogen levels and WGTT of the three volunteers from groups A and B who failed to complete were similar to those who did. The data gained from their completed sections were included in the analysis.

Ingestion of senna and loperamide caused the intended changes in WGTT, with corresponding changes in stool output, stool form and defecatory frequency (Table 1). With wheat bran, stool form and frequency clearly changed appropriately, whereas WGTT tended to decrease and stool output to increase. Stool pH changed only with senna, when it fell from 7.2 (s.d. 0.5) to 6.8 (s.d. 0.7) ($P = 0.04$); stool β-glucuronidase activity was unaffected by any intervention.

The serum concentrations of total and non-protein-bound oestradiol failed to change with any of the interventions (Table 2). However oestrone, both total and non-protein bound fractions, fell in those taking senna. Decreases in the concentrations of oestrone

© Cancer Research Campaign 1997

**Table 2** Oestradiol, sex hormone-binding globulin and albumin concentrations at the start and end of each interventional period (geometric mean, 95% CI, 95% CI of the ratio of the geometric means and *P*-value)

| | Baseline | 95% CI | Active | 95% CI | 95% CI of the ratio | *P*-value |
|---|---|---|---|---|---|---|
| Oestradiol (pmol $l^{-1}$) | | | | | | |
| Wheat bran | 281.1 | (212.7, 371.5) | 262.0 | (199.3, 344.5) | (–1.31, 1.14) | 0.48 |
| Senna | 261.1 | (194.2, 351.1) | 225.8 | (175.0, 291.3) | (–1.50, 1.13) | 0.28 |
| Loperamide | 233.1 | (184.8, 293.9) | 248.6 | (189.5, 326.2) | (–1.25, 1.43) | 0.64 |
| Calculated non-protein-bound oestradiol (pmol $l^{-1}$) | | | | | | |
| Wheat bran | 5.5 | (4.2, 7.1) | 5.1 | (4.0, 6.6) | (–1.33, 1.16) | 0.49 |
| Senna | 5.2 | (4.1, 6.7) | 4.5 | (3.6, 5.6) | (–1.52, 1.11) | 0.22 |
| Loperamide | 4.5 | (3.7, 6.2) | 4.8 | (3.7, 6.2) | (–1.35, 1.37) | 0.96 |

| | Baseline | IQ range | Active | IQ range | 95% CI of difference | *P*-value |
|---|---|---|---|---|---|---|
| Sex hormone-binding globulin (nmol $l^{-1}$) | | | | | | |
| Wheat bran | 56.0 | (39.5, 71.8) | 51.5 | (41.0, 77.3) | (–6.5, 2.5) | 0.31 |
| Senna | 44.0 | (36.0, 65.0) | 44.0 | (38.0, 73.0) | (–2.0, 5.0) | 0.41 |
| Loperamide | 49.5 | (43.3, 63.5) | 52.5 | (40.0, 68.0) | (–4.0, 6.5) | 0.86 |
| Albumin (g $l^{-1}$) | | | | | | |
| Wheat bran | 44.0 | (42.8, 45.0) | 44.5 | (40.0, 48.0) | (0.0, 2.0) | 0.09 |
| Senna | 44.0 | (43.0, 46.0) | 44.0 | (43.0, 46.0) | (–1.0, 1.5) | 0.93 |
| Loperamide | 42.0 | (41.0, 45.0) | 43.5 | (42.0, 47.5) | (–0.5, 3.5) | 0.12 |

**Table 3** Serum oestrone and oestrone sulphate concentrations at the start and end of each interventional period (geometric mean, 95% CI, 95% CI of the ratio of the geometric means and *P*-value)

| | Baseline | 95% CI | Active | 95% CI | 95% CI of ratio | *P*-value |
|---|---|---|---|---|---|---|
| Oestrone (pmol $l^{-1}$) | | | | | | |
| Wheat bran | 239.9 | (193.9, 296.8) | 246.1 | (203.7, 297.4) | (–1.13, 1.19) | 0.72 |
| Senna | 252.1 | (211.3, 300.7) | 205.9 | (179.5, 236.2) | (1.05, 1.42) | 0.01 |
| Loperamide | 219.3 | (185.7, 258.9) | 239.9 | (192.6, 298.8) | (–1.07, 1.28) | 0.24 |
| Calculated non-protein-bound oestrone (pmol $l^{-1}$) | | | | | | |
| Wheat bran | 11.0 | (8.7, 13.0) | 11.0 | (9.0, 13.0) | (–1.14, 1.20) | 0.74 |
| Senna | 11.0 | (9.8, 13.0) | 9.2 | (8.2, 10.0) | (1.06, 1.44) | 0.01 |
| Loperamide | 6.7 | (5.6, 8.0) | 6.8 | (5.6, 8.3) | (–1.16, 1.21) | 0.78 |
| Oestrone sulphate (pmol $l^{-1}$) | | | | | | |
| Wheat bran | 1744.6 | (1412.5, 2154.3) | 1523.3 | (1256.3, 1846.7) | (1.00, 1.31) | 0.04 |
| Senna | 1833.2 | (1529.0, 2198.0) | 1647.4 | (1391.6, 1950.7) | (1.00, 1.24) | 0.04 |
| Loperamide | 1640.6 | (1352.1, 1990.7) | 1819.7 | (1421.7, 2329.2) | (–1.05, 1.28) | 0.18 |

sulphate were seen with wheat bran as well as senna (Table 3). No changes in the oestrogens were observed in volunteers taking loperamide (Tables 2 and 3). No changes were seen in SHBG and albumin concentrations during any intervention (Table 2).

There was no significant difference between dietary intakes, specifically total fibre, NSP, or fat, at the start, middle and end of each interventional period. The mean baseline intake for the three groups was 16.3 g $day^{-1}$ (s.d. 4.3) for fibre and 11.1 g $day^{-1}$ (s.d. 2.8) for NSP. No volunteers reported a change in their diet. Volunteers taking wheat bran consumed a mean of 19.8 g $day^{-1}$ (s.d. 7.1), providing 9.1 g $day^{-1}$ of dietary fibre (Southgate, 1977) and 8.1 g $day^{-1}$ of NSP.

### DISCUSSION

The subjects in this study can be considered sufficiently representative. Their baseline dietary intake of fibre and its fractions was greater than that reported for American adults (Anderson et al, 1989), but similar to that of English women [21.5 g $day^{-1}$ (Emmett et al, 1993) and 18.6 g $day^{-1}$ (Gregory et al, 1990)]. Their median whole-gut transit time (64 h) was very similar to that (62 h) of a large group of healthy premenopausal women (Probert et al, 1995) and their baseline stool outputs were in the range of values (100–200 g $day^{-1}$) reported for UK women (Wyman et al, 1978; Cummings et al, 1992). The intended changes in bowel function occurred with all three supplements, although the transit time change just escaped significance with wheat bran.

Women of reproductive age were studied because epidemiological evidence links increased exposure to oestrone and oestradiol to their subsequent development of breast cancer. Moreover, their oestrogen concentrations are higher than after the menopause, permitting more precise measurement and therefore enhanced detection of changes in response to treatment. If our hypothesis that intestinal transit speed is a determinant of serum oestrogen concentration is correct, then this influence will occur in both pre- and post-menopausal women. Samples for assay were collected early in the follicular phase of the ovarian cycle. Alternative sampling times such as at mid-cycle or in mid-luteal phase can only be identified reliably in individuals after subsequent menstruation. Use of such times would have required many more samples to ensure the exact location of the peak levels.

© Cancer Research Campaign 1997

**Table 4** Percentage change in early follicular serum oestrogens in this study and that of Goldin et al (1994)

| | Goldin et al High fibre | This study | | |
|---|---|---|---|---|
| | | Wheat bran | Senna | Loperamide |
| Non-protein bound oestradiol | −4.9 | −7.1 | −17.5 | 0.0 |
| Oestradiol | −11.0[a] | −7.5 | −15.7 | +6.7 |
| Oestrone | −4.7 | +2.5 | −22.4[a] | +8.8 |
| Oestrone sulphate | −17.1[a] | −14.8[a] | −12.2[a] | +8.8 |

[a]*P*-value < 0.05.

The major finding of this study is that in subjects taking senna serum concentrations of oestrone and oestrone sulphate fell and on wheat bran there was a decreased concentration of oestrone sulphate. These findings support our hypothesis that faster intestinal transit decreases the absorption of oestrogens thereby reducing the exposure of the body tissues to oestrogens. It is likely that the effect of senna is mediated via the colon as this laxative has little or no effect on small bowel transit (Marcus and Heaton, 1986). The observation that the slower WGTT brought about by loperamide did not result in higher serum oestrogen concentrations may imply that reabsorption is already maximal in British women under normal conditions. The apparent lack of effect of bran on WGTT may be misleading. In 7 of the 18 volunteers there was no decrease in WGTT and hence a non-significant result for the group as a whole. Such variability in the response to bran has been reported before (Eastwood *et al.*, 1973). A laxative dose may not be achieved if bran causes bloating or excess flatus.

The present findings may be compared with those of Rose *et al* (1991), who doubled the daily fibre intake (from 15 to 30 g day[1]) of 62 women by administration of additional wheat, oats or corn bran for two menstrual cycles. Consumption of wheat bran, but not oats or corn, decreased the serum concentrations of oestradiol and oestrone in the early luteal phase. On combining dietary records and oestrogen measurements before and after 2 months of interventions, a negative correlation was found between dietary fibre intake and serum oestrone concentrations. However, subjects consuming wheat bran also increased their energy, carbohydrate and fat intake, and this may have influenced serum oestrogens.

A different protocol was used by Goldin *et al* (1994), who measured early follicular phase serum oestrogen concentrations in women on a high-fat/low-fibre diet (fat 40% of calories, fibre 12 g $day^{-1}$) and on a low-fat/high-fibre diet (fat 20–25% of calories, fibre 40 g $day^{-1}$) at constant calorie intake. From multiple regression analysis these authors concluded that an increase in fibre intake decreased serum concentration of oestradiol and oestrone sulphate but was without effect on oestrone.

The ability of dietary fibre to alter intestinal transit time depends on the type of fibre and how it has been processed. In the study by Rose *et al.* (1991), no transit or defecatory data were collected, but oats and corn probably have less effect than wheat on intestinal transit (Cummings, 1993). The fact that corn and oats bran had no effect on serum oestrogens despite their ability to bind oestrogens under experimental conditions (Shultz and Howie, 1986; Arts et al, 1991) suggests that the binding of oestrogens to bran is not an important mechanism in the reduction of serum oestrogen concentrations. The use of processed and cooked wheat bran by Rose et al (1991) and Goldin et al. (1994) complicates any comparison with the present study, in which only raw bran, a more effective laxative (Wyman et al. 1976), was used. Despite these caveats, taken together the three studies suggest that manipulation of colonic function alters serum oestrogen levels (for example see Table 4).

It is likely that the effects on serum oestrogens are brought about by interference with the enterohepatic circulation. Rose et al, (1991) invoked reduction of bacterial β-glucuronidase activity and/or binding of oestrogens to explain their findings, whereas Goldin et al (1994) offered no explanation for theirs. In our study there was no change in stool β-glucuronidase activity with any of the dietary supplements used. Ingestion of senna was, however, accompanied by a significant decrease in stool pH, and if this reflected the pH of the colonic lumen then in vivo β-glucuronidase activity might well have been reduced, leading to diminished availability of unconjugated oestrogens for reabsorption. The explanation we prefer is that, by speeding up transit, senna reduced the time available for the hydrolysis and absorption of oestrogens from the colon.

Which oestrogens are involved in the aetiology and promotion of oestrogen-dependent diseases is not known. Moreover, the biological importance of the changes in serum oestrone and oestrone sulphate observed by us remains to be proved but, if they are important, speeding up colonic transit might reduce the risk of breast cancer in Western populations whose intestinal transit time tends to be slow compared with that of rural Third World populations (Burkitt et al, 1972).

### ACKNOWLEDGEMENTS

We thank Reg Fletcher, Kathryn O'Sullivan, Peter Cripps and Tony Hughes for help in designing the study and the statistical interpretation of the data. The technical assistance of Carol Symes and the staff of the SAS Centre for Steroid Hormones is gratefully acknowledged. This work was supported by a generous grant from the Kellogg Company of Great Britain.

### REFERENCES

Adlercreutz H, Martin F, Lehtinen T, Tikkanen MJ and Pulkkinen MO (1977) Effect of ampicillin administration on plasma conjugated and unconjugated estrogen and progesterone levels in pregnancy. *Am J Obstet Gynecol* **128**: 266–271

Anderson JW, Bridges SR, Tietyen J and Gustafson NJ (1989) Dietary fiber content of a simulated American diet and selected research diets. *Am J Clin Nutr* **49**: 352–357

Armstrong B and Doll R (1975) Environmental factors and the cancer incidence and mortality in different countries, with special reference to dietary practices. *Int J Cancer* **15**: 617–631

Armstrong BK, Brown JB, Clarke HT, Crooke DK, Hänthel R, Masarei JR and Ratajczak T (1981) Diet and reproductive hormones: A study of vegetarians and nonvegetarian postmenopausal women. *J Natl Cancer Inst* **67**: 761–767

Arts CJM, Govers CAR, Berg HVD, Wolters MGE, Leeuwen PV and Thussen JHH (1991) In vitro binding of oestrogens by dietary fiber and the in vivo apparent digestibility tested in pigs. *J Steroid Biochem Molec Biol* **38**: 621–628

© Cancer Research Campaign 1997

Buell P (1973) Changing incidence of breast cancer in Japanese–American women. *J Natl Cancer Inst* **51**: 1479–1483

Burkitt DP, Walker ARP and Painter NS (1972) Effect of dietary fibre on stools and transit times and its role in the causation of disease. *Lancet* **ii**: 1408–1412

Cummings JH (1993) The effect of dietary fibre on fecal weight and composition. In *CRC Handbook of Dietary Fiber in Human Nutrition*, Spiller, GA (ed.), pp. 263–349. CRC Press: Boca Raton

Cummings JH, Bingham SA, Heaton KW and Eastwood MA (1992) Faecal weight, colon cancer risk, and dietary intake of nonstarch polysaccharides (dietary fibre). *Gastroenterology* **103**: 1783–1789

Drasar BS and Irving D (1973) Environmental factors and cancer of the colon and breast. *Br J Cancer* **27**: 167–172

Eastwood MA, Kirkpatric JR, Mitchell WD, Bone A and Hamilton T (1973) Effects of dietary supplements of wheat bran and cellulose on faeces and bowel function. *Br Med J* **4**: 392–394

Emmett PM, Symes CL and Heaton KW (1993) Dietary intake and sources of non-starch polysaccharide in English men and women. *Eur J Clin Nutr* **47**: 20–30

Englyst HN, Bingham SA, Runswick SA, Collinson E and Cummings JH (1988) Dietary fibre (non-starch polysaccharides) in fruit, vegtables and nuts. *Gastroenterology* **1**: 247–286

Englyst HN, Bingham SA, Runswick SA, Collinson E and Cummings JH (1989) Dietary fibre (non-starch polysaccharides) in cereal products. *J Hum Nutr Diet* **2**: 253–271

Feng W, Marshall R, Lewis-Barned NJ and Goulding A (1993) Low follicular oestrogen levels in New Zealand women consuming high fibre: a risk factor for osteopenia? *N Z Med J* **106**: 319–322

Goldin BR and Gorbach SL (1976) The relationship between diet and rat fecal bacterial enzymes implicated in colon cancer. *J Natl Cancer Inst* **57**: 371–375

Goldin BR, Adlercreutz H, Gorbach SL, Warram JH, Dwyer JT, Swenson L and Woods MN (1982) Estrogen excretion patterns and plasma levels in vegetarian and omnivorous women. *N Engl J Med* **307**: 1542–1547

Goldin BR, Woods MN, Spiegelman DL, Longcope C, Morrill-Labrode A, Dwyer JT, Gualtiere LJ, Hertzmark E and Gorbach SL (1994) The effect of dietary fat and fiber on serum estrogen concentrations in premenopausal women under controlled dietary conditions. *Cancer* **74**: 1125–1131

Gorbach SL and Goldin BR (1987) Diet and the excretion and enterohepatic cycling of estrogens. *Prev Med* **16**: 525–531

Gregory J, Foster K, Tyler H and Wiseman M (1990) *The Dietary and Nutritional Survey of British Adults*. HMSO: London

Howe GR, Hirohata T, Hislop TG, Iscovich JM, Yuan JM, Katsouyanni K, Lubin F, Marubini E, Modan B, Rohan T, Toniolo P and Shunzhang Y (1990) Dietary factors and the risk of breast cancer, combined analysis of 12 case control studies. *J Natl Cancer Inst* **82**: 561–569

Katsouyanni K, Trichopoulos D, Boyle E, Xirouchaki E, Trichopoulou A, Lisseos B, Vasilaros S and MacMahon B (1986) Diet and breast cancer: A case controlled study in Greece. *Am J Cancer* **38**: 815–820

Kim DH, Kang HJ, Kim SW and Kobashi K (1992) pH-inducable β-glucosidase and β-glucuronidase of intestinal bacteria. *Chem Pharm Bull* **40**: 1667–1669

Lea AJ (1966) Dietary factors associated with death-rates from certain neoplasms in man. *Lancet* **2**: 332–333

Lewis SJ, Bolton C and Heaton KW (1996) Lack of influence of intestinal transit on oxidative status in premenopausal women. *Eur J Clin Nutr* **50**: 565–568

Lubin F, Wax Y and Modan B (1986) Fat, protein and fibre in breast cancer aetiology: A case-controlled study. *J Natl Cancer Inst* **77**: 605–611

McGarrigle HHG and Lachelin GCL (1983) Oestrone, oestradiol and oestriol glucosiduronates and sulphates in human puerperal plasma and milk. *J Steroid Biochem* **18**: 607–611

Mallet AK, Rowland IR and Bearne CA (1985) Modification of rat caecal microbial biotransformation activities by dietary saccharin. *Toxicology* **36**: 253–262

Marcus SN and Heaton KW (1986) Intestinal transit, deoxycholic acid and the cholesterol saturation of bile: three inter-related factors. *Gut* **27**: 550–558

Martin F, Peltonen J, Laatikainen T, Pulkkinen M and Adlercreutz H (1975) Excretion of progesterone metabolites and estriol in faeces from pregnant women during ampicillin administration. *J Steroid Biochem* **6**: 1339–1346

Miller AB (1977) Role of nutrition in the etiology of breast cancer. *Cancer* **39**: 2704–2708

Neale G (1983) Effects of fibre on the entero-hepatic circulation of oestradiol. In *Dietry Fibre Symposium*, Vol. 26, pp. 206. Fibre in Human and Animal Nutrition Bulletin. Royal Society of New Zealand: New Zealand

O'Donnell LJD, Virjee J and Heaton KW (1990) Detection of pseudodiarrhoea by simple clinical assessment of intestinal transit rate. *Br Med J* **300**: 439–440

Paul AA and Southgate DAT (1978) *McCance and Widdowson's The Composition of Foods*. Elsevier/North-Holland Biomedical Press: Amsterdam

Probert CSJ, Emmett PM and Heaton KW (1993) Intestinal transit time in the population calculated from self made observations of defecation. *J Epidemiol Comm Health* **47**: 331–333

Probert CSJ, Emmett PM and Heaton KW (1995) Some determinants of whole gut transit-time: a population-based study. *Q J Med* **88**: 311–315

Reddy BS, Engle A, Katsifis S, Bartram HP, Perrino P and Mahan C (1989) Biochemical epidemiology of colon cancer: effect of types of dietary fiber on fecal mutagens, acid, and neutral sterols in healthy subjects. *Cancer Res* **49**: 4629–4635

Rose DP, Goldman M, Connolly JM and Strong LE (1991) High-fibre diet reduces serum estrogen concentrations in premenopausal women. *Am J Clin Nutr* **54**: 520–525

Shultz TD and Howie BJ (1986) In vitro binding of steroid hormones by natural and purified fibres. *Nutr Cancer* **8**: 141–147

Shultz TD and Leklem JE (1983) Nutrient intake and hormonal status of premenopausal vegetarian seventh-day adventists and premenopausal nonvegetarians. *Nutr Cancer* **4**: 247–257

Southgate DA (1977) The definition and analysis of dietary fiber. *Nutrl Rev* **35**: 31–37

Speight AC, Hancock KW and Oakey RE (1979) Non-protein bound oestrogens in plasma and urinary excretion of unconjugated oestrogens in men. *Clin Endocrinol* **10**: 329–341

Staszewski J and Haenszel W (1965) Cancer mortality among the Polish-born in the United States. *J Natl Cancer Inst* **35**: 291–297

Whittaker JA, Cawood ML and Oakey RE (1992) A method for the determination of sex hormone binding globulin using Concanavalin A-sepharose. *Ann Clin Biochem* **29**: 168–171

Willman K and Pulkkinen MO (1971) Reduced maternal plasma and urinary estriol during ampicillin treatment. *Am J Obstet Gynaecol* **109**: 893–896

Wyman JB, Heaton KW, Manning AP and Wicks ACB (1976) The effect on intestinal transit and the feces of raw and cooked bran in different doses. *Am J Clin Nutr* **29**: 1474–1479

Wyman JB, Heaton KW, Manning AP and Wicks ACB (1978) Variability of colonic function in healthy subjects. *Gut* **19**: 146–150

Zaridze D, Lifanova Y, Maximovitch D, Day NE and Duffy SW (1991) Diet, alcohol consumption and reproductive factors in a case-control study of breast cancer in Moscow. *Int J Cancer* **48**: 493–501

 © Cancer Research Campaign 1997